# Differential expression of TNFR1 (CD120a) and TNFR2 (CD120b) on subpopulations of human monocytes

**DOI:** 10.1186/1476-9255-9-38

**Published:** 2012-10-05

**Authors:** Daniëlle Hijdra, Adriane DM Vorselaars, Jan C Grutters, Anke ME Claessen, Ger T Rijkers

**Affiliations:** 1 Department of Medical Microbiology and Immunology, St. Antonius Hospital, Nieuwegein, The Netherlands; 2 Centre for Interstitial Lung Diseases, Department of Pulmonology, St. Antonius Hospital, Nieuwegein, The Netherlands; 3 Division Heart & Lungs, University Medical Center Utrecht, Utrecht, The Netherlands; 4 Department of Sciences, Roosevelt Academy, Middelburg, The Netherlands

**Keywords:** Monocytes, CD120a, CD120b, TNF receptor, CD14, CD16, HLA-DR, Peripheral blood, Sarcoidosis

## Abstract

**Background:**

Three subpopulations of monocytes can be distinguished in human blood: classical (CD14^++^CD16^−^), intermediate (CD14^++^CD16^+^), and nonclassical (CD14^+^CD16^++^). CD16 expressing monocytes are expanded in patients with sarcoidosis and in various other inflammatory diseases. In sarcoidosis, it is unclear whether either intermediate, nonclassical or both CD16 expressing monocytes are responsible for this increase. Data relating to the monocyte subpopulations is receiving increasing attention, but the expression of TNF receptors on these subpopulations has not been studied thus far. The aim of this study was to determine frequencies of monocyte subpopulations and their expression of TNFR1 and TNFR2 in both sarcoidosis patients and healthy controls.

**Methods:**

Peripheral blood cells of sarcoidosis patients and healthy controls were stained for the markers HLA-DR, CD14, CD16, CD120a and CD120b. Cells were measured on a FACSCalibur and analyzed with FlowJo. We used Student’s *t*-test and a parametric One-way ANOVA for statistical analysis.

**Results:**

Sarcoidosis patients had a significant higher frequency of intermediate monocytes than healthy controls. Significant differences in TNF receptor expression were found between the monocyte subpopulations, both in sarcoidosis patients as well as in healthy controls: intermediates expressed more TNFR1 than classicals and nonclassicals and nonclassicals expressed more TNFR2 than intermediates, whereas intermediates showed higher expression than classicals.

**Conclusions:**

In both sarcoidosis patients and healthy controls intermediate monocytes show the highest expression level of TNFR1 among monocyte subpopulations and nonclassical monocytes show the highest expression level of TNFR2. These findings, as wells as the higher frequency of intermediate monocytes in sarcoidosis patients, provide evidence for the existence of two functionally-distinct CD16 expressing monocyte subpopulations.

## Background

Monocytes originate from myeloid progenitors in the bone marrow, circulate in the blood for up to three days, and then enter peripheral tissues where they differentiate into macrophages or dendritic cells [1].

Human monocytes can be divided into three subpopulations: classical, intermediate, and nonclassical. This subdivision is based on the expression levels of the lipopolysaccharide (LPS) coreceptor CD14 and the Fcγ receptor III CD16. Classical monocytes, the major population of monocytes, are CD14^++^CD16^−^. The minor population of CD16 expressing monocytes are further subdivided in intermediate monocytes (CD14^++^CD16^+^) and nonclassical monocytes (CD14^+^CD16^++^) [2]. Recently, monocyte subpopulations have been extensively investigated. In addition to CD14 and CD16, also other markers, such as HLA-DR, CCR2 and CCR5, can distinguish the three human monocyte subpopulations [3,4].

An imbalance in the relative proportion of CD16 expressing monocytes has been found in a variety of immune mediated diseases such as rheumatoid arthritis, diabetes, atherosclerosis, bacterial and HIV infections (reviewed by Ziegler-Heitbrock [5]). An increase in the relative number of CD16 expressing monocytes was found in newly diagnosed sarcoidosis patients, suggesting an activated state of the monocytes. This activated state might be an indicator for disease activity [6,7]. These investigations have not discriminated between the two CD16 expressing monocyte subpopulations. Recently, it has been shown in patients with rheumatoid arthritis [8] and severe asthma [9] that intermediate monocytes are responsible for the increase in CD16 expressing monocytes. So far, this has not been investigated in patients with sarcoidosis.

Sarcoidosis is a systemic, granulomatous disease of unknown origin that primarily affects the lungs. It is a disease of all races and ethnic groups with varying incidence throughout the world [10-12]. The highest incidence of sarcoidosis in Europe has been reported from Sweden: 24 cases per 100,000 [13]. In The Netherlands, the incidence of sarcoidosis is estimated to be 20 cases per 100,000 [14]. In the United States, the incidence rate in black people is 36 cases per 100,000 compared to 11 cases per 100,000 in white people [15]. The most important feature of sarcoidosis is the formation of non-caseating granulomas [10-12].

In the human lung, tumor necrosis factor (TNF) is mainly produced by alveolar macrophages and thought to play a major role in the development of the granulomatous inflammation. The release of TNF is increased in the lung of patients with pulmonary sarcoidosis [16,17]. Therefore TNF is an attractive target for immunotherapy in sarcoidosis.

TNF exerts its function by binding to and signaling via two different receptors. TNFR1 (CD120a) is constitutively, but in low levels, expressed on nearly all nucleated cell types. TNFR2 (CD120b) is inducible and expressed by cells of myeloid lineage, peripheral T cells and alveolar lymphocytes and macrophages [18-23]. TNFR1 activates the caspase family, which induce cell death. TNFR2 signalling leads to NF-ĸB transcription and can induce proliferation, differentiation, cytokine production and even apoptosis. When TNFR1 and TNFR2 are co-expressed on the same cells, intracellular cross-talk between the receptors may occur [24]. Both TNF receptors can be enzymatically cleaved from the cell surface and form soluble TNF receptors. Soluble TNF receptors can neutralize TNF and clear it from the circulation and act as a TNF antagonist, but can also prolong its bioactivity by binding TNF and thus stabilize the trimeric structure [22]. Membrane-bound TNF binds more strongly to TNFR2 than soluble TNF does. It may be that TNFR2 only becomes fully activated with membrane-bound TNF [21].

There are a few reports of TNFR1 and TNFR2 expression on peripheral blood cells. Approximately 25% of the peripheral blood lymphocytes in healthy subjects is CD4^+^TNFR2^+^. Interestingly, in sarcoidosis the percentage of CD4^+^TNFR2^+^ cells is increased, especially in patients in remission or with stable disease (respectively 35% and 33%), which may indicate a role in down regulation of a cell-mediated immune response [23]. FoxP3^+^ T reg cells expressed the highest levels of TNFR2 among subsets of human peripheral blood CD4^+^ T cells and were able to shed large amounts of sTNFR2, suggesting an important role for T reg cells in suppressing TNF [25]. It is attractive to speculate about a similar role for one of the subpopulations of human monocytes. However, the expression of TNFR1 and TNFR2 on monocyte subpopulations has not been studied thus far.

The aim of the present study was to determine frequencies of the three monocyte subpopulations in peripheral blood of patients with sarcoidosis compared to healthy controls and the relative expression of TNFR1 and TNFR2 on these subpopulations.

## Methods

### Patients and healthy controls

Thirty-eight patients (11 female, 27 male; median age 48 years, range 22–70 years) with proven sarcoidosis were included in the present study. For this study, all sarcoidosis patients were grouped as one, independent of severity of the disease and independent of use of medication. Thirteen healthy volunteers (4 female, 9 male; median age 50 years, range 27–58 years) were used as controls. Peripheral blood was collected in sodium heparin tubes. The study was approved by the medical ethical evaluation committee of the St Antonius Hospital in Nieuwegein, The Netherlands.

### Antibodies and flow cytometry

Anti-HLA-DR FITC and anti-CD16 PE were obtained from BD Biosciences (San Diego, CA, USA). Anti-CD14 PerCP-eFluor710 was from eBioscience (San Diego, CA, USA). Anti-CD120a APC (TNFR1) and anti-CD120b APC (TNFR2) were from R&D Systems (Minneapolis, MN, USA). After staining, flow cytometry data were acquired on a FACSCalibur (BD Biosciences) and analyzed using FlowJo software (Tree Star, Ashland, OR, USA).

Monocytes were gated based on FSC x SSC and SSC x HLA-DR^+^ expression. Subsequently, monocytes were subdivided in three populations based on CD14 and CD16 expression pattern. We used fluorescence minus one (FMO) staining as control for the TNF receptor staining by substracting the MFI of the FMO control of the MFI of the TNF receptor.

### Statistical analysis

Data are expressed as mean ± SEM. Comparison of two groups of data (patients versus controls) was performed by a two-tailed Student’s *t*-test and comparison of three groups of data (subpopulations of monocytes) was performed by a parametric One-way ANOVA, both by using GraphPad Prism version 5.0 for Windows (GraphPad Software, San Diego, CA, USA). Differences were considered statistically significant at *P* values of 0.05 or less.

## Results and discussion

### Monocytes subpopulations

**Figure 1:**
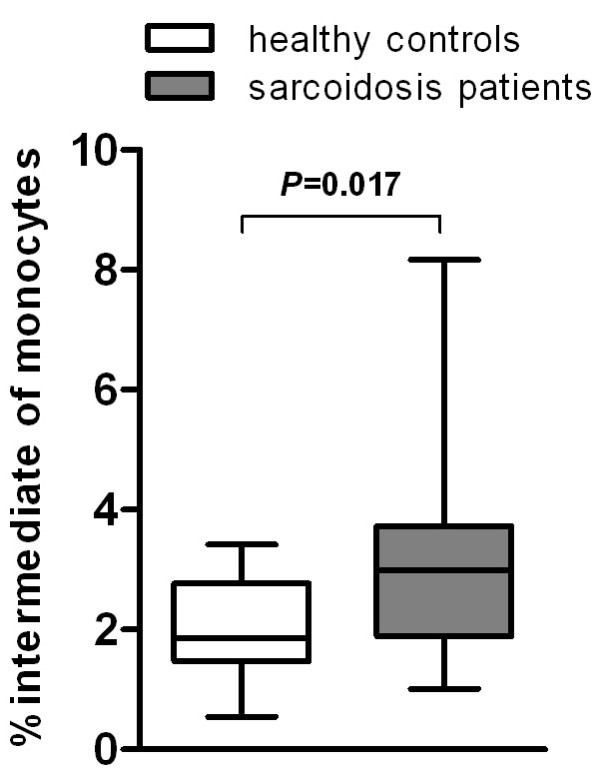
**Sarcoidosis patients show a higher frequency of intermediate monocytes than healthy controls.** Percentage of intermediates within the monocyte population of patients with sarcoidosis (n = 38) and healthy controls (n = 13). Data are presented as box plots, where the boxes represent the 25^th^ to 75^th^ percentiles, the lines within the boxes represent the median, and the lines outside the boxes represent the 10^th^ and 90^th^ percentiles.

The frequency of classical, intermediate, and nonclassical monocyte subpopulations was analyzed in 38 sarcoidosis patients and 13 healthy controls, based on HLA-DR, CD14 and CD16 expression pattern. In this study, classical monocytes accounted for 60–90% of SSC x HLA-DR^+^ gated monocytes, intermediate monocytes for about 2–5% and nonclassical monocytes for 5–25%, both in sarcoidosis patients as well as in healthy controls. Others (reviewed by Ziegler-Heitbrock [5]) have found increased or decreased relative numbers of CD16 expressing monocytes in a variety of inflammatory and/or infectious diseases in man. In most of these studies nonclassical and intermediate monocytes were grouped together as one subpopulation. Rossol *et al.* reported that increased frequencies of CD16 expressing monocytes in rheumatoid arthritis were due to an increase in intermediates [8]. Moniuszko *et al.* reported similar findings for severe asthma [9].We also found a higher frequency of intermediate monocytes in sarcoidosis patients (3.08% ± 0.25%) than in healthy controls (1.98% ± 0.22%; *P* = 0.017; Figure 1), while nonclassical monocytes were not increased (data not shown). It should be stressed that CD16 expressing monocytes should be considered as two different populations.

**Figure 2:**
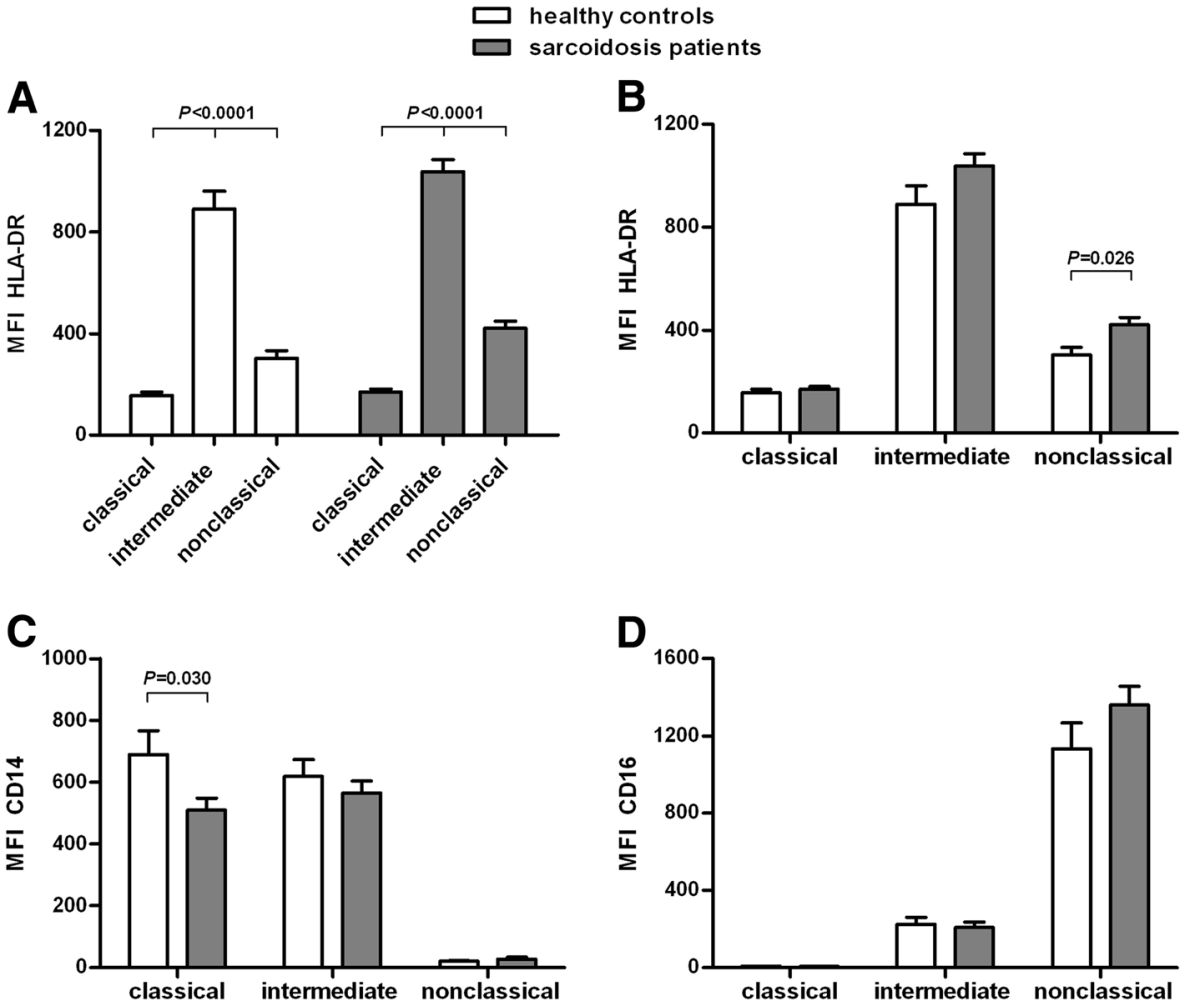
**Differential expression of HLA-DR, CD14, CD16 on monocyte subpopulations in sarcoidosis patients and healthy controls.** Median fluorescence intensity (MFI) of HLA-DR (**A**,**B**), CD14 (**C**) and CD16 (**D**) on classical (CD14^++^CD16^−^), intermediate (CD14^++^CD16^+^) and nonclassical (CD14^+^CD16^++^) monocytes in sarcoidosis patients (n = 38) and in healthy controls (n = 13). Please note that, for sake of clarity, the data in panel A are regrouped in panel B to allow better comparison between the monocyte subpopulations. Values are expressed as mean ± SEM. All three markers were significantly different between the three monocyte subpopulations. Between sarcoidosis patients and healthy controls, nonclassical monocytes showed significant different levels of HLA-DR expression and classical monocytes showed significant different levels of CD14 expression. Comparison of three groups of data (subpopulations of monocytes) was performed by a parametric One-way ANOVA and comparison of two groups of data (patients versus controls) was performed by a two-tailed Student’s *t*-test.

All three monocyte subpopulations differed significantly in HLA-DR expression, both in sarcoidosis patients as well as in healthy controls. Intermediates showed the highest expression of HLA-DR, followed by nonclassicals (*P* < 0.0001; Figure 2A). This differential expression of HLA-DR on monocyte subpopulations previously has been described for patients with rheumatoid arthritis [8] and for healthy controls [3].

We found significant differences in HLA-DR and CD14 expression between patients with sarcoidosis and healthy controls. Nonclassical monocytes of sarcoidosis patients expressed a higher level of HLA-DR than nonclassicals of controls (*P* = 0.026; Figure 2B). Classical monocytes of sarcoidosis patients expressed a lower level of CD14 than classicals of healthy controls (*P* = 0.030; Figure 2C). Intermediate monocytes of sarcoidosis patients tended to have a higher level of HLA-DR and a lower level of CD14 than intermediates of healthy controls (Figures 2B and 2C). Nonclassical and intermediate monocytes of sarcoidosis patients tended to have a higher level of CD16 than nonclassicals and intermediates of controls (Figure 2D).

### TNF receptors

**Figure 3:**
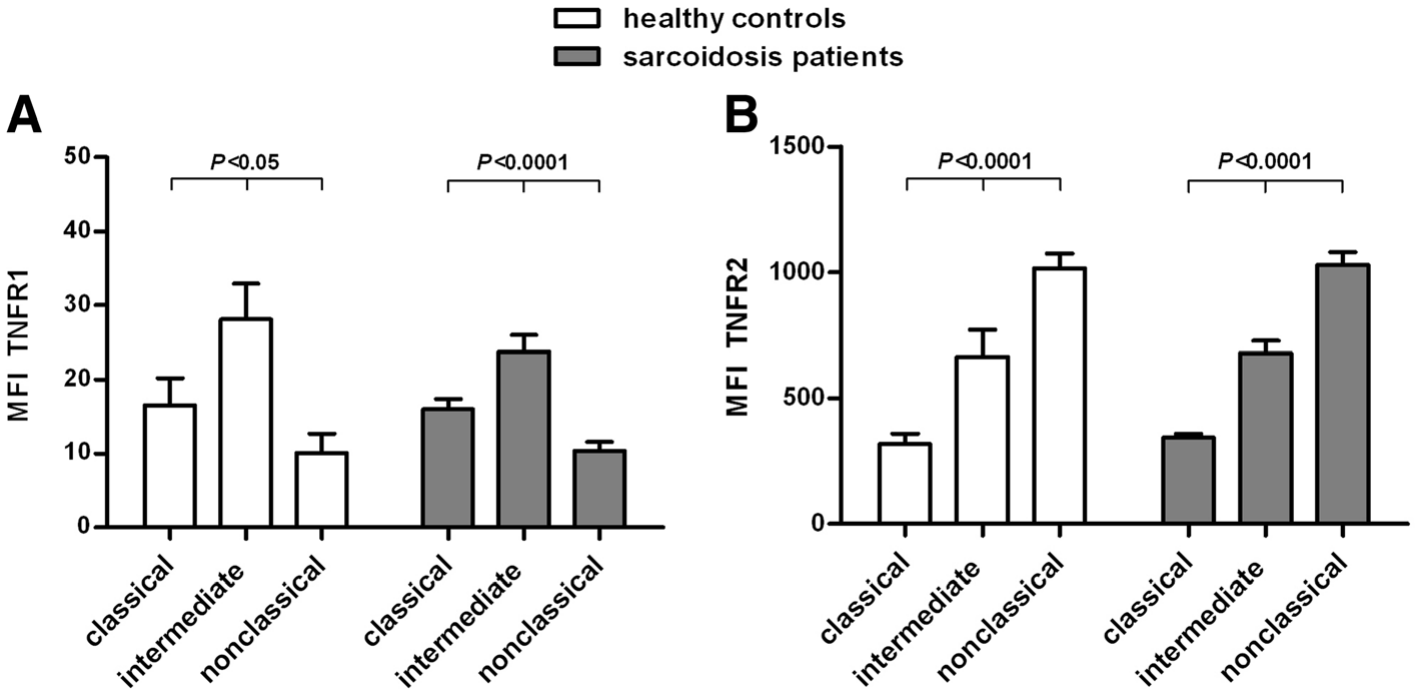
**Differential expression of TNFR1 and TNFR2 on monocyte subpopulations in sarcoidosis patients and healthy controls.** Median fluorescence intensity (MFI) of TNFR1 (**A**) and TNFR2 (**B**) on classical (CD14^++^CD16^−^), intermediate (CD14^++^CD16^+^) and nonclassical (CD14^+^CD16^++^) monocytes in sarcoidosis patients (TNFR1 n = 18, TNFR2 n = 38) and in healthy controls (TNFR1 n = 5, TNFR2 n = 13) minus the MFI of the fluorescence minus one (FMO) control. Values are expressed as mean ± SEM. Both markers were significantly different between the three monocyte subpopulations. All monocytes expressed TNFR1, but intermediates had a higher expression of TNFR1 than classicals and nonclassicals. All monocytes also expressed TNFR2, but nonclassicals had a higher expression of TNFR2 than intermediates and a much higher expression than classicals. Comparison of the three subpopulations of monocytes was performed by a parametric One-way ANOVA.

TNF is a major cytokine regulating the activity of monocytes and therefore we have extended the phenotypic characterization of monocyte subpopulations for expression of TNF receptors. Although no differences were found between patients and controls, analysis of TNFR1 and TNFR2 revealed differential expression on the monocyte subpopulations, both in sarcoidosis patients as well as in healthy controls. All monocytes expressed TNFR1, but intermediates showed a higher expression of TNFR1 than classicals and nonclassicals (*P* < 0.05; Figure 3A). All monocytes also expressed TNFR2, but the three subpopulations showed major differences in TNFR2 expression (*P* < 0.0001; Figure 3B). Nonclassical monocytes expressed the highest levels of TNFR2. Intermediates expressed less TNFR2 than nonclassicals, but more than classicals. Although classical monocytes expressed the lowest levels of TNFR2, values were still largely positive compared to a fluorescence minus one (FMO) control.

## Conclusions

Subpopulations of monocytes not only express different levels of CD14, CD16 and HLA-DR, but also different levels of TNFR1 and TNFR2. This finding is new and emphasizes the diversity and potential different functions of the three monocyte subpopulations. Frequencies of intermediate monocytes are increased in sarcoidosis patients, suggesting an activated state of the monocytes in this disease. This finding may be of especial relevance because intermediate monocytes express the highest level of HLA-DR among the monocyte subpopulations. This activated state might be a novel indicator for disease activity. Intermediate monocytes also express the highest level of TNFR1 among the monocyte subpopulations, while nonclassical monocytes, the other CD16 expressing monocyte subpopulation, express the highest level of TNFR2. This data supports the evidence of two functionally-distinct CD16 expressing monocyte subpopulations.

HLA-DR may be a valuable marker to monitor the disease course of sarcoidosis patients. Intermediate and nonclassical monocytes plus their TNF receptor expression pattern might be relevant to monitor for sarcoidosis patients who are going to be treated with anti-TNF (Remicade), as well as for other patients on anti-TNF therapy, such as those suffering from rheumatoid arthritis, psoriasis or Crohn’s disease. Prospective monitoring of these patients should demonstrate whether particular monocyte subpopulations display differential sensitivity to this form of treatment and whether this correlates with the clinical response.

## Abbreviations

ANOVA: Analysis of variance; FACS: Fluorescence activated cell sorter; FMO: Fluorescence minus one; HIV: Human immunodeficiency virus; LPS: Lipopolysaccharide; MFI: Median fluorescence intensity; NF-ĸB: Nuclear factor kappa-light-chain-enhancer of activated B cells; SEM: Standard error of the mean; SSC: Side scatter; TNF: Tumor necrosis factor alpha; TNFR1: Tumor necrosis factor receptor 1; TNFR2: Tumor necrosis factor receptor 2.

## Competing interests

The authors declare that they have no competing interests.

## Authors’ contributions

DH performed experiments, designed the research protocols, analyzed data and wrote the manuscript. AV selected patients and collected blood samples. JG selected patients and designed the research protocols. AC designed the research protocols. GR designed the research protocols. All authors interpreted data, edited the manuscript, read and approved the final manuscript.
